# Repositioning Fenofibrate to Reactivate p53 and Reprogram the Tumor-Immune Microenvironment in HPV+ Head and Neck Squamous Cell Carcinoma

**DOI:** 10.3390/cancers14020282

**Published:** 2022-01-07

**Authors:** W. Quinn O’Neill, Xiujie Xie, Shanying Gui, Heping Yu, Jacqueline Davenport, Thomas Cartwright, Marta Storl-Desmond, Esther Ryu, Ernest R. Chan, Shufen Cao, Pingfu Fu, Theodoros N. Teknos, Quintin Pan

**Affiliations:** 1 University Hospitals Seidman Cancer Center, Cleveland, OH 44106, USA; wqo@case.edu (W.Q.O.); guishanying@hotmail.com (S.G.); Theodoros.Teknos@UHhospitals.org (T.N.T.); 2 Department of Otolaryngology—Head and Neck Surgery, University Hospitals, Case Western Reserve University School of Medicine, Cleveland, OH 44106, USA; hxy44@case.edu (H.Y.); jacqueline.davenport@case.edu (J.D.); thomas.cartwright@case.edu (T.C.); marta.storl-desmond@case.edu (M.S.-D.); esther.ryu@case.edu (E.R.); 3 Department of Otolaryngology—Head and Neck Surgery, The Ohio State University Wexner Medical Center, Columbus, OH 43210, USA; xx8df@virginia.edu; 4 Case Comprehensive Cancer Center, Case Western Reserve University School of Medicine, Cleveland, OH 44106, USA; ricky.chan@case.edu (E.R.C.); shufen.cao@case.edu (S.C.); pingfu.fu@case.edu (P.F.)

**Keywords:** anti-cancer therapy, human papillomavirus, hypoxia, PD-1, PD-L1, tumor microenvironment

## Abstract

**Simple Summary:**

A critical need for optimal management of human papillomavirus-associated head and neck squamous cell carcinoma (HPV+ HNSCC) patients is the development of therapeutic strategies to exploit the inherent vulnerabilities of this unique disease. We identified fenofibrate, an FDA-approved drug, as a potent anti-cancer agent for HPV+ HNSCC. Fenofibrate induced the accumulation of the p53 tumor suppressor and re-programmed the tumor microenvironment to drive immune cell infiltration. We provide compelling evidence to reposition fenofibrate as a single agent or in combination with standard therapies for the HPV+ HNSCC setting.

**Abstract:**

Human papillomavirus-associated head and neck squamous cell carcinoma (HPV+ HNSCC) is recognized as a distinct disease with unique etiology and clinical features. Current standard of care therapeutic modalities are identical for HPV+ and HPV− HNSCC and thus, there remains an opportunity to develop innovative pharmacologic approaches to exploit the inherent vulnerabilities of HPV+ HNSCC. In this study, using an inducible HPVE6E7 knockdown system, we found that HPV+ HNSCC cells are addicted to HPVE6E7, such that loss of these viral oncogenes impaired tumorigenicity in vitro and in vivo. A number of druggable pathways, including PPAR and Wnt, were modulated in response to HPVE6E7 loss. Fenofibrate showed significant anti-proliferative effects in a panel of HPV+ cancer cell lines. Additionally, fenofibrate impaired tumor growth as monotherapy and potentiated the activity of cisplatin in a pre-clinical HPV+ animal model. Systemic fenofibrate treatment induced p53 protein accumulation, and surprisingly, re-programmed the tumor-immune microenvironment to drive immune cell infiltration. Since fenofibrate is FDA-approved with a favorable long-term safety record, repositioning of this drug, as a single agent or in combination with cisplatin or checkpoint blockade, for the HPV+ HNSCC setting should be prioritized.

## 1. Introduction

Human papillomavirus-associated head and neck squamous cell carcinoma (HPV+ HNSCC) is recognized as an etiologically, epidemiologically, and clinically distinct disease. High-risk HPVs exhibit a tropism for the lymphoid tissue of the oropharynx and malignant transformation is believed to occur in the reticular epithelium of the tonsillar crypts [1,2]. The obscurity of this anatomic site complicates visual detection of early-stage lesions and consequently, these patients most often present with nodal metastasis, sometimes with an undetectable primary tumor [3]. In the United States, more than 10,000 people are diagnosed annually with HPV+ HNSCC [4]. This number has been increasing steadily in recent decades, and the trend is expected to continue until at least 2060, even anticipating the potential preventive effects of HPV vaccination [5].

Despite the many distinctive features of HPV+ HNSCC, current treatment options for HNSCC, are the same irrespective of HPV status. Standard of care remains surgery and/or platinum-based chemoradiotherapy; however, these treatments are associated with high morbidity [6]. Curative surgery for HNSCC can leave patients with significant disfigurement and masticatory dysfunction, while platinum-based chemoradiation is associated with debilitating health issues, including dysphagia, xerostomia, and ototoxicity [6,7,8]. These morbidities may be especially burdensome for patients with HPV+ HNSCC, who tend to be younger than their HPV− counterparts and face reduced quality of life for many years after oncologic treatment [9,10]. HPV+ HNSCC patients also tend to respond very favorably to treatment, so there is growing interest in treatment de-escalation strategies for this patient group. Unfortunately, none of these de-escalation approaches has yet proven to maintain similar efficacy as standard of care paradigms.

On a molecular level, HPV+ HNSCCs are distinguished by wildtype p53, a major tumor suppressor protein that is usually mutated in HPV− HNSCCs, as well as in a majority of cancers from other anatomical sites [11]. Gain-of-function mutations of p53 are common in HPV− HNSCC and bestow new functions to drive cancer initiation and progression. In HPV+ HNSCC, p53 is predominantly wildtype but its tumor suppressive actions are compromised by the viral oncoprotein, E6, through two distinct mechanisms. HPVE6 interacts with the E3 ubiquitin ligase, E6AP, to promote proteasomal degradation of wildtype p53 [12,13,14]. In addition, HPVE6 associates with p300 to block p53 acetylation, impairing both the stability of p53 and its transcriptional activity [15,16,17,18,19,20].

As our group and others have shown, HPV+ cancers are addicted to the sustained expression of HPVE6E7 oncoproteins [21,22,23,24,25,26,27,28,29,30]. The necessity of these viral oncoproteins to the survival of HPV+ cancer cells represents a biological vulnerability that can be exploited for the development of effective targeted therapies for this patient population. At the present time, there are no clinically approved therapies to directly target either of these viral oncoproteins or their major downstream targets, p53 and pRb. Moreover, de novo development of new targeted therapies is an expensive and time-consuming undertaking. Therefore, as an alternative approach, we sought to identify signaling pathways that are co-opted by HPVE6E7 which could be effectively targeted by re-positioning drugs that are already FDA-approved for other indications. We selected several such drugs for further investigation and ultimately revealed fenofibrate, an FDA-approved anti-hyperlipidemia drug, to be a promising candidate that bridges a translational gap toward a well-tolerated, effective treatment tailored for HPV+ malignancies.

## 2. Materials and Methods

### 2.1. Cell Lines

CaSki and CAL27 cell lines were purchased from ATCC (Manass, VA, USA) and the HEK293T cell line was purchased from Thermo Fisher Scientific (Waltham, MA, USA). UMSCC47 was provided by Dr. Thomas Carey, University of Michigan, UD-SCC2 was obtained from Henning Bier, of Heinrich-Heine University, Dusseldorf, Germany, and UPCI:SCC090 was provided by Dr. Susanne Gollin of the University of Pittsburgh. CAL27, UD-SCC2, UMSCC47, UPCI:SCC090, and HEK293T cell lines were grown in Dulbecco’s modified Eagle’s medium (DMEM) containing 10% fetal bovine serum (FBS) and 1% Pen-Strep (100 mg/mL streptomycin, and 100 U/mL penicillin). CaSki cells were cultured in RPMI-1640 medium with 10% FBS and 1% Pen-Strep. All cell lines were authenticated using STR profile analysis and were maintained in a humidified atmosphere of 5% CO_2_ at 37 °C.

### 2.2. Generation of Inducible shRNA-HPV16E6E7 Cell Lines

The HPV16E6E7 transcript has three alternate splicing patterns. In order to target all splicing transcripts, three shRNA oligonucleotides were selected, one with a target upstream of the spliced region (shRNA3) and two with downstream targets (shRNA4 and shRNA-5) [31]. These oligonucleotides were synthesized and cloned into the inducible tetracycline-on pLV-RNAi vector (BioSettia, San Diego, CA, USA). HEK293T cells were plated in 100-mm plates and allowed to attach overnight. The following day, at a 50–60% confluency, the cells were transfected with the HPV16E6E7 shRNA plasmid using packaging plasmids psPAX2 (Addgene, San Diego, CA, USA) and pMD2.G (Addgene), and the medium was replaced with Opti-MEM (Thermo Fisher Scientific) after 6–8 h. Lentivirus-containing media were harvested at 3 days and passed through a 0.45-μm filter (Millipore, Billerica, MA, USA) to generate lentiviral stocks. Viral transduction was accomplished by incubating cells in viral medium for 48 h, followed by 72 h of puromycin selection (Thermo Fisher Scientific) to generate polyclonal populations. To induce HPV16E6E7-targeting shRNA, cells were treated with doxycycline at 1000 ng/mL.

### 2.3. Immunoblot

Whole cell lysates were mixed with Laemmli loading buffer, boiled, separated by SDS–PAGE, and transferred to a nitrocellulose membrane. Subsequently, immunoblot analyses were performed using antibodies specific to HPV16E6 (abcam, Cambridge, MA, USA), HPV16E7 (CERVIMAX, Vienna, Austria), HIF1α (Cell Signaling, Danvers, MA, USA), PD-L1 (abcam), or GADPH (Millipore).

### 2.4. Tumorsphere Formation Assay

Cells were collected and seeded in serum-free keratinocyte medium supplemented with epidermal growth factor, basic fibroblast growth factor, insulin and hydrocortisone in low-attachment plates (Corning Incorporated, Corning, NY, USA). Tumorsphere formation efficiency was calculated by dividing the number of tumorspheres (≥50 µm in diameter) formed in 7 days by the initial number of cells seeded. Tumorsphere diameter was measured using the NIS-Elements software (Nikon Instruments, Melville, NY, USA).

### 2.5. Colony Formation Assay

Cells were plated at a density of 300 cells per well in six-well plates and incubated for 10 days in their respective conditions. Colonies were fixed with 10% cold methanol, stained with 0.1% crystal violet in 20% methanol, washed, and air dried. Colonies were counted manually.

### 2.6. Cell Cycle and Apoptosis

Cells were plated in serum-free medium and incubated overnight for cell cycle synchronization. For inducible shRNA-HPV16E6E7 cells, the medium was changed to complete growth medium with or without doxycycline. For fenofibrate treatment, the medium was changed to complete medium with dimethyl sulfoxide (DMSO; Thermo Fisher Scientific) or 100 µM fenofibrate (Selleckchem, Houston, TX, USA). The proportion of cells at each stage of the cell cycle was evaluated by flow cytometric analysis of propidium iodide fluorescence (ab139418; abcam). Apoptosis was assessed using a Violet Annexin V/Dead Cell Apoptosis Kit (A35136; Thermo Fisher Scientific) and analyzed by flow cytometry.

### 2.7. Senescence

Cells were seeded at a density of 100,000 cells per well in six-well plates and allowed to attach overnight. Cells were treated with DMSO or 100 µM fenofibrate for 24 h and β-galactosidase staining was performed according to manufacturer’s instructions (Senescence β-galactosidase Staining Kit 9860; Cell Signaling Technology, Danvers, MA, USA). Stained cells were visualized with brightfield microscopy at 200× total magnification and a minimum of five independent fields were analyzed per condition. Cells were counted manually, and positive cells were expressed as a percent of total cells per field.

### 2.8. Cell Proliferation Analysis

Cell proliferation was assessed using the Incucyte Live Cell Imaging System (Sartorius, Gottingen, Germany). Each cell line was seeded in 96-well plates at a density of 2000 cells per well in a 75 µL volume of complete medium. Following overnight attachment, an additional 25 µL volume of complete medium containing a 4× concentration of each drug dose was added to each well. Respective drug doses were first prepared in DMSO at 400× concentration and then diluted 1/100 in complete medium, so that all wells received the same volume of DMSO.

### 2.9. In Vivo Efficacy Models

All animal experiments were reviewed and approved by the Case Western Reserve University IACUC under protocol# 2018-0013. For the chemoprevention model, UMSCC47-shRNA4 (1 × 10^6^) cells were implanted subcutaneously into the flanks of 7- to 8-week-old, female athymic nude mice and fed control diet or doxycycline-containing diet (200 mg/kg doxycycline hyclate; Envigo, East Millstone, NJ, USA) ad libitum. For the therapeutic model, UMSCC47-shRNA4 (1 × 10^6^) cells were implanted subcutaneously into the flanks of 7- to 8-week-old, female athymic nude mice and fed control diet until day 28. Subsequently, tumor-bearing mice were randomly assigned to continue on the control diet or switch to doxycycline-containing diet ad libitum. For the evaluation of fenofibrate as monotherapy and in combination with cisplatin, UMSCC47 (1 × 10^6^) cells were delivered by subcutaneous injection into the flanks of 8-week-old, female athymic nude mice. When tumors reached a mean volume of approximately 60 mm^3^, mice were assigned to four treatment groups, each with a similar mean tumor volume (*n* = 7 per group). Groups received control (vehicle), fenofibrate, cisplatin, or combination treatments. Fenofibrate (200 mg/kg) was delivered by gavage once daily five times per week in a 100 µL volume of 10% DMSO/0.5% carboxymethylcellulose. The fenofibrate suspension was first prepared in DMSO and then combined with 0.5% carboxymethylcellulose (Sigma Aldrich, St. Louis, MO, USA). The vehicle control used was 10% DMSO/0.5% carboxymethylcellulose. Cisplatin (2 mg/kg; dissolved in 0.9% sterile saline; Sigma Aldrich) was delivered once weekly by intraperitoneal injection. Tumors were measured daily using a digital caliper and tumor volume was calculated using the formula: volume = 1/2 (length × width^2^).

### 2.10. Histopathology and p53 Immunohistochemistry

Tumors were harvested and fixed in 10% neutral buffered formalin. Tissue embedding, sectioning, hematoxylin- and eosin-staining, and immunohistochemical staining for p53 (ab32389; abcam) was performed by the Case CCC Tissue Resource Core. Tumor cells with intense nuclear staining were considered p53+. Given the scattered pattern of positive cells, data were represented as averages of counts per high-powered field (HPF; 200×). Only fields that were comprised of tumor cells were considered for analysis; stromal regions and necrotic areas were excluded. All slides were reviewed and evaluated for p53 staining by a board-certified oral pathologist (WQO).

### 2.11. Statistical Analysis

Student’s *t*-tests and chi-squared tests were performed in R. A mixed-effect model was used to estimate the tumor growth rate over time for the fenofibrate in vivo efficacy study. Temporal profiles of tumor growth by treatment were visualized by scatter plot superimposed with a LOWESS smoother (locally weighted scatterplot smoothing). In the mixed model, we assume that measurements made during follow-up for the same mouse are correlated. Unstructured covariance was used for inference. All tests were two-sided and *p*-values less than 0.05 were considered statistically significant.

## 3. Results

### 3.1. Inducible Silencing of HPV16E6E7 Promotes a Pleotropic Anti-Cancer Effect in HPV16+ SCC

**Figure 1:**
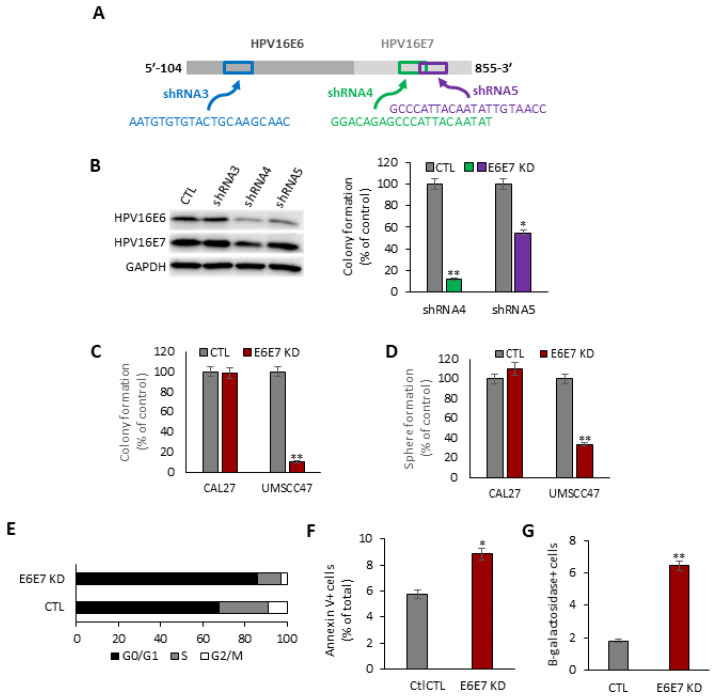
Inducible HPV16E6E7 knockdown drives a pleotropic anti-cancer effect in HPV16+ SCC cells. (**A**) Design of short hairpin RNA (shRNA) constructs targeting HPV16E6 or HPV16E7. (**B**) Validation of shRNA constructs to target HPV16E6 and HPV16E7, and suppress colony formation. Immunoblot for HPV16E6 and HPV16E7 (**left**). Uncropped immunoblots are available in File S1. Colony formation assay (**right**). Number of colonies as a percentage of control (CTL) count for HPV16E6E7 knockdown (E6E7 KD) induced by shRNA constructs 4 and 5. * *p* < 0.05; ** *p* < 0.01. (**C**) Colony formation assay. Number of colonies as a percentage of CTL. ** *p* < 0.01. (**D**) Tumorsphere formation assay. The number of tumorspheres is shown as a percentage of the CTL count. ** *p* < 0.01. (**E**) Cell cycle analysis. Black, gray, and white bars indicate the percentage of the total population in each cell cycle phase for CTL and HPV16E6E7 KD cells. (**F**) Apoptosis assay. Percentage of CTL and HPV16E6E7 UMSCC47 cells that are annexin V+ as measured by flow cytometry. * *p* < 0.05. (**G**) Senenscence assay. The percentage of CTL and HPV16E6E7 UMSCC47 cells that are B-galactosidase positive. ** *p* < 0.01.

Three doxycycline-inducible shRNA vectors were constructed to target distinct locations of the HPV16E6E7 transcript (Figure 1A). UMSCC47, an HPV16+ HNSCC cell line, was separately transduced with each HPV16E6E7-targeting vector and polyclonal populations were selected. shRNA4 reduced HPV16E6 and HPV16E7 levels, whereas shRNA5 preferentially diminished HPV16E6 levels (Figure 1B). Compared to the uninduced parental cells, colony formation was reduced by 88.0% (*p* < 0.01) by shRNA4 and by 45.0% (*p* < 0.05) by shRNA5 (Figure 1B). Since shRNA4 was the most active, this construct was used to target HPV16E6E7 in all subsequent experiments.

Our inducible shRNA4 HPV16E6E7 silencing platform was used in HPV− Cal27 to serve as a negative control. Colony formation was severely compromised by 89.5% (*p* < 0.01) in HPV16+ UMSCC47 cells following doxycycline induction (Figure 1C). A tumorsphere formation assay was performed to evaluate the effect of HPV16E6E7 silencing on the cancer initiating cell (CIC) population. As shown in (Figure 1D), HPV16E6E7 knockdown in UMSCC47 cells reduced tumorsphere formation efficiency by 66.7% (*p* < 0.01). HPV16E6E7 silencing decreased the proportion of UMSCC47 cells in S phase by 54.5% (*p* < 0.01) and increased the proportion of cells in G0/G1 phase by 26.8% (*p* < 0.05) (Figure 1E). Moreover, HPV16E6E7 knockdown promoted apoptosis by 53.9% (*p* < 0.05) (Figure 1F) and cellular senescence by 255% (*p* < 0.0001) (Figure 1G). In contrast to the HPV16+ SCC cell lines, induction of HPV16E6E7 shRNA had no effect on cell proliferation, clonogenic survival, or the CIC population in HPV− Cal27 cells, indicating that our shRNA4 inducible system had minimal off-target effects.

### 3.2. Inducible Silencing of HPV16E6E7 Inhibits In Vivo Tumorigenicity in HPV16+ HNSCC

**Figure 2:**
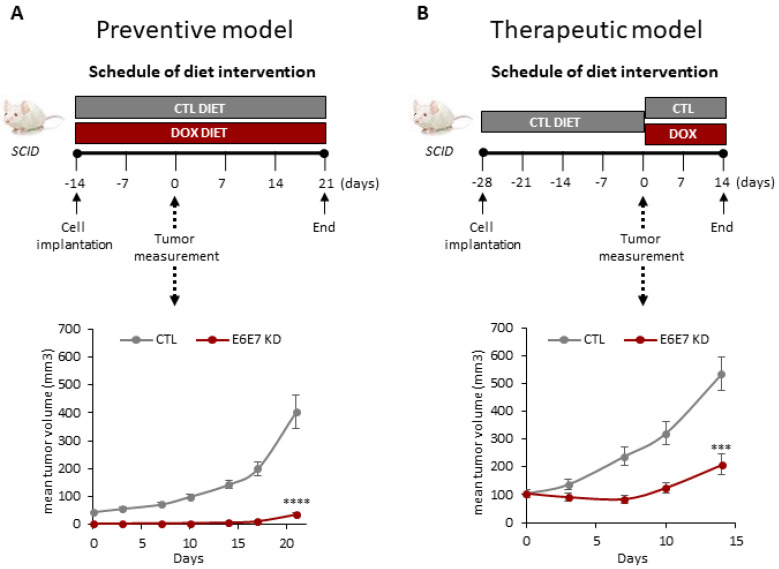
Inducible HPV16E6E7 knockdown inhibits the in vivo tumorigenicity of HPV16+ SCC. (**A**) Chemoprevention model. UMSCC47-shRNA4 (1 × 10^6^) cells were implanted subcutaneously into the flanks of 7- to 8-week-old, female athymic nude mice and fed control diet or doxycycline-containing diet (200 mg/kg doxycycline hyclate) ad libitum. **** *p* < 0.0001. (**B**) Therapeutic model. UMSCC47-shRNA4 (1 × 10^6^) cells were implanted subcutaneously into the flanks of 7- to 8-week-old, female athymic nude mice and fed a control diet until the development of established tumors. Subsequently, animals were randomized to continue to receive the control diet or doxycycline-containing diet (200 mg/kg doxycycline hyclate) ad libitum. *** *p* < 0.001.

Two different animal models, chemoprevention and therapeutic, were used to assess the consequence of HPV16E6E7 silencing on HPV16+ HNSCC cells in vivo. For the chemoprevention model, athymic nude mice were randomly assigned to receive control diet or doxycycline-containing diet immediately after polyclonal UMSCC47-shRNA4 cells (1 × 10^6^) were implanted (Figure 2A). All seven mice receiving the control diet had measurable tumors by day 14, compared to none of the mice receiving doxycycline. Impairment of tumorigenesis was sustained for the duration of the study, with tumor volume diminished by 90.7% (*p* < 0.0001) in the doxycycline-containing diet mice at day 35. Mean tumor volume was 37.4 ± 9.3 mm^3^ for the HPV16E6E7 knockdown group and 403.6 ± 150.0 mm^3^ for the control group. For the therapeutic model, all the animals received the control diet until day 28, when mice were randomly assigned to continue on the control diet or switch to the doxycycline-containing diet (Figure 2B). Doxycycline induction suppressed tumor volume by 60.9% (*p* < 0.001); mean tumor volume was 208.9 ± 87.6 mm^3^ for the HPV16E6E7 knockdown group and 534.3 ± 138.9 mm^3^ for the control group. These findings are consistent with our in vitro results and confirm that HPV16+ SCC cells are addicted to HPVE6E7 to maintain tumorigenicity.

**Figure 3:**
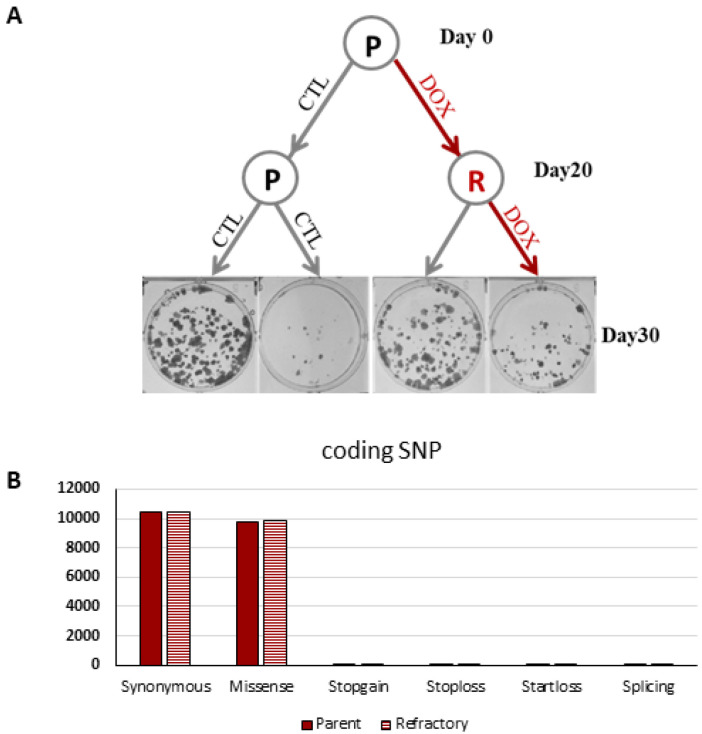
Inducible HPV16E6E7 knockdown refractory cells have lower copies of HPV16E6E7 shRNA. UMSCC47-shRNA4 cells were unstimulated or stimulated with doxycycline for 20 continuous days to generate a paired parental and refractory cell line. (**A**) Colony formation assay. Parental and refractory cells were plated and treated with control or doxycycline for 10 days. Representative cell culture plates are shown for each experimental condition. (**B**) Whole exome sequencing. Exonic SNVs were unchanged between UMSCC47-shRNA parental and refractory cells.

Tumor growth was not completely ablated in these two experimental tumor growth models suggesting that a subset of HPV16+ tumor cells may be refractory to HPVE6E7 silencing. To directly address this question, parental UMSCC47-shRNA4 cells were exposed to vehicle or doxycycline continuously for 20 days to generate matched HPVE6E7 sensitive and refractory polyclonal populations. HPVE6E7 refractory cells had lower integrated copies of HPVE6E7 shRNA in their genome than HPVE6E7 sensitive cells (Figure 3). Moreover, exonic single nucleotide variant frequency and profile was comparable between these paired cell lines. These findings provide evidence that a threshold level of HPVE6E7 is necessary for tumor maintenance and de novo resistance to HPVE6E7 silencing is likely a rare event.

### 3.3. Inducible Silencing of HPV16E6E7 Reveals Temporal Modulation of Key Signal Transduction Pathways

**Figure 4:**
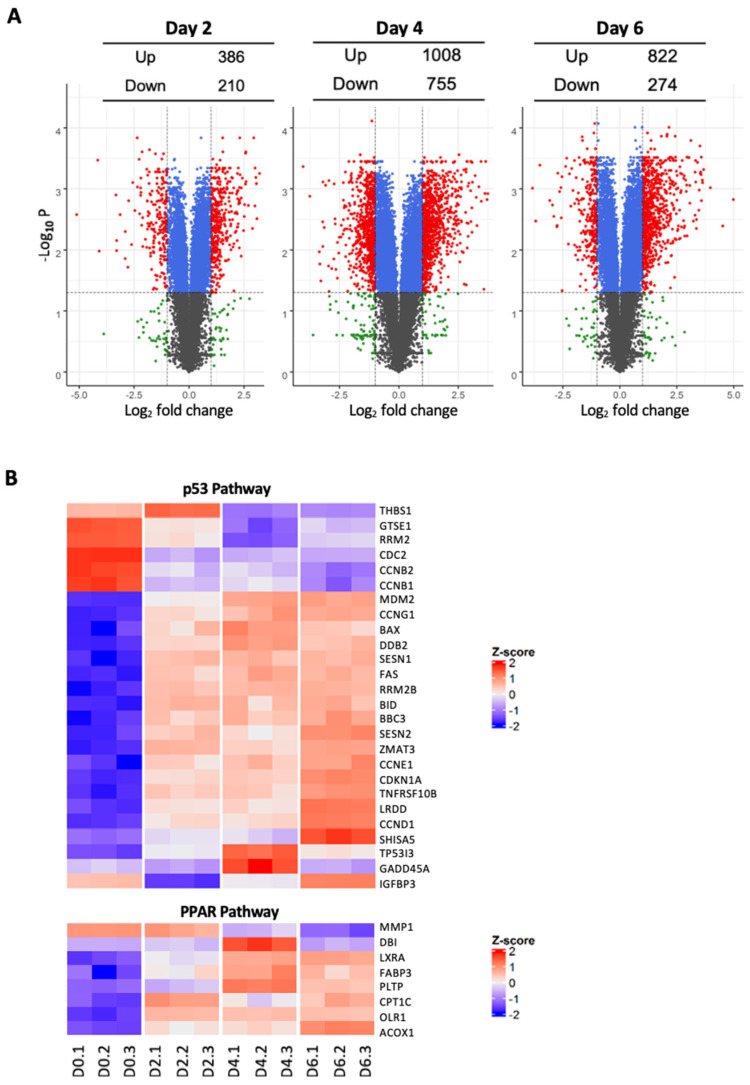
Inducible HPV16E6E7 knockdown alters the transcriptomic signature of HPV16+ SCC cells. (**A**) Volcano plot. Total RNA was extracted from UMSCC47-shRNA4 cells at day 0 and at days 2, 4, and 6 after doxycycline induction using TRIZol and processed for RNA-Seq. Transcriptomic and KEGG pathway analyses were performed with the GAGE package in R. (**B**) Heat map of representative genes in the p53 and PPAR pathways.

To examine the global transcriptomic effect of HPV16E6E7 depletion, RNA-Seq was performed in UMSCC47 cells at baseline (day 0) and after doxycycline induction for 2, 4, and 6 days. Principal component analysis (PCA) showed that the first two principal components were sufficient to capture 98.2% (90.2% + 8.0%) of the gene expression variation indicating that the majority of differences across these experimental conditions can be explained and represented by PCA. Differential gene expression (DGE) analysis revealed a dynamic reshaping of the transcriptome following HPVE6E7 knockdown (Figure 4). Compared to baseline (day 0), 1235 (833 up-regulated and 402 down-regulated) genes were differentially regulated at day 2 and increased to 2911 (1674 up-regulated and 1247 down-regulated) genes at day 4.

Pathway analysis was performed to investigate the key signaling pathways altered following HPVE6E7 repression. Gene set enrichment analysis showed 28 and 29 KEGG canonical pathways significantly up-regulated and down-regulated, respectively (FDR adjusted *p* < 0.05) (Table S1). In line with our phenotypic results, the cell cycle pathway was strongly inhibited at day 2 and day 4 post HPVE6E7 silencing. As would be expected following repression of HPVE6, the p53 signaling pathway was among the top up-regulated pathways in days 2 and 4. Interestingly, the apoptosis pathway was significantly enhanced on day 2, but this change was transient and reversed by day 4, indicating that the increase in apoptosis is an early event following HPVE6E7 loss

### 3.4. Fenofibrate Partially Recapitulates HPV16E6E7 Silencing in HPV+ SCC

**Figure 5:**
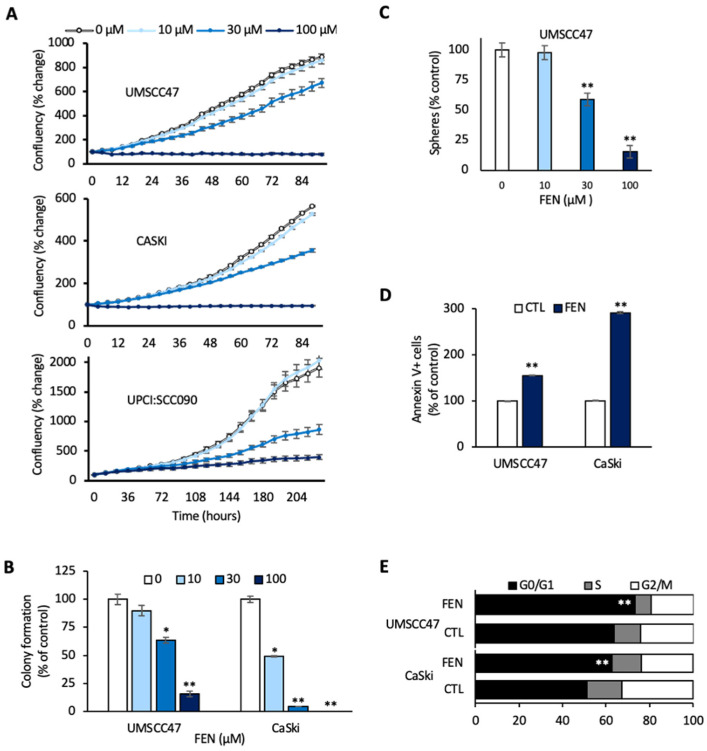
Fenofibrate induces a global anti-cancer effect. (**A**) Cell proliferation. UMSCC47, UPCI:SCC090, and CaSki cell lines were treated with control or fenofibrate. Cell proliferation was measured using the IncuCyte live cell imaging system. Data are represented as mean ± SEM. (**B**) Colony formation assay. Colony counts are shown as a percentage of control for the indicated doses of fenofibrate. * *p* < 0.05; ** *p* < 0.01. (**C**) Tumorsphere formation assay. Tumorsphere counts are shown as a percentage of control for the indicated doses of fenofibrate. ** *p* < 0.01. (**D**) Apoptosis assay. Percentage of Annexin V+ cells following control or fenofibrate treatment, ** *p* < 0.01. (**E**) Cell cycle analysis. Black, gray, and white bars indicate the percentage of the total population in each cell cycle phase in response to control or fenofibrate treatment, ** *p* < 0.01.

Two of the pathways modulated following HPVE6E7 knockdown were selected for validation to confirm the robustness of our platform. Wnt and PPAR pathways were chosen due to the availability of molecules to modulate these pathways with high specificity. Fenofibrate, a PPARα agonist, and FH535, a Wnt inhibitor, resulted in dose-dependent impairment of cellular proliferation in HPV16+ UMSCC47 and UPCI:SCC090 HNSCC cell lines; complete ablation of cell growth was observed at 100 µM and 30 µM doses of fenofibrate and FH535, respectively (Figure 5A and Figure S1). 

We decided to focus on fenofibrate since it is an FDA-approved drug that has demonstrated a favorable toxicity profile, even with daily dosing over an extended period. Fenofibrate (100 µM) reduced clonogenic survival by 84% (*p* < 0.001) and tumorsphere formation by 86% (*p* < 0.001) in UMSCC47 cells. CaSki cells showed complete abrogation of colony formation at the same dose (*p* < 0.001) (Figure 5B,C). In response to fenofibrate, UMSCC47 cells had a 55% (*p* < 0.01) increase in apoptotic cells and a 15% (*p* < 0.01) increase in the proportion of cells in the G1 phase, while CaSki cells showed a dramatic 190% increase in apoptotic cells (*p* < 0.0001) and a 22% increase in the fraction of cells in G1 phase (*p* < 0.001) (Figure 5D,E). Cellular senescence was similar between control and fenofibrate-treated UMSCC47 or CaSki cells (data not shown). Together, these results demonstrate that the induction of cell cycle arrest and apoptosis contribute to the anti-proliferative effects of fenofibrate treatment in HPV+ SCC.

### 3.5. Fenofibrate Ablates In Vivo Tumor Growth in a Pre-Clinical Model of HPV+ HNSCC

**Figure 6:**
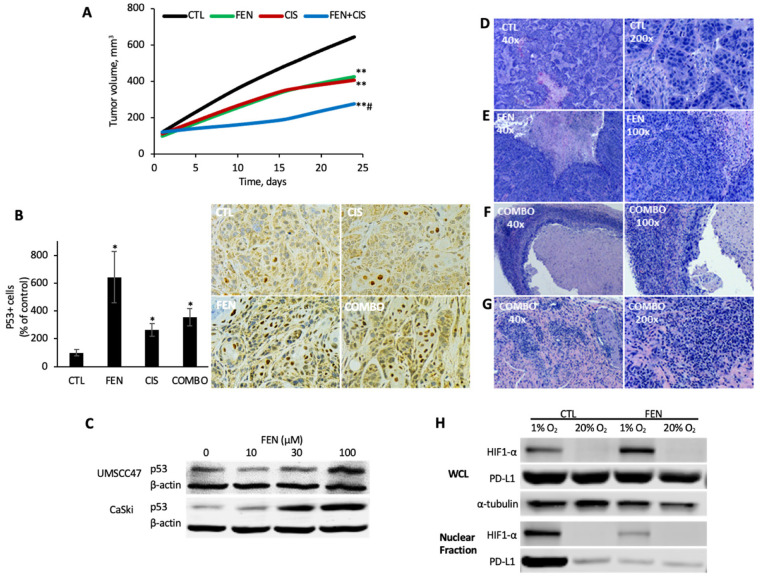
Fenofibrate inhibits in vivo tumorigenicity, promotes p53 accumulation, and reprograms the tumor-immune microenvironment. (**A**) UMSCC47 xenograft model. UMSCC47 cells were implanted into the flanks of athymic nude mice and tumors were allowed to develop without intervention. Tumor-bearing mice (~100 cm^3^) were randomized to four treatment arms: control (CTL), fenofibrate (FEN; 200 mg/kg PO, 5 times/week), cisplatin (CIS; 2 mg/kg IP; once weekly), or fenofibrate + cisplatin (COMBO). ** FEN or CIS vs. CTL, *p* < 0.001 or COMBO vs. CTL, *p* = 0.0001; # COMBO vs. FENO or CIS, *p* = 0.0001. (**B**) p53 immunohistochemistry. p53 levels were analyzed in harvested UMSCC47 xenograft tumors and tumor cells with strong nuclear staining was scored as p53+. A representative IHC image for each experimental condition is shown (200×). * *p* < 0.01. (**C**) p53 levels. Immunoblot for p53 protein levels following control or fenofibrate treatment in UMSCC47 cells. Uncropped immunoblots are available in File S1. (**D**–**G**) Hematoxylin- and eosin-stained tumor sections. Representative tumor sections illustrating notable morphological features, at low power (40×; left) and higher power magnification (100–200×; right). (**H**) HIF1α and PD-L1 levels. Immunoblot for nuclear HIF1α and PD-L1 levels under normoxic (20% O_2_) or hypoxic (1% O_2_) conditions with control or fenofibrate treatment in UMSCC47 cells. Uncropped immunoblots are available in File S1.

In a HPV16+ xenograft model, fenofibrate was as active as cisplatin and both single-agent modalities impaired tumor growth by ~50% (*p* < 0.01, *n* = 7) (Figure 6A). The combination regimen of fenofibrate and cisplatin was superior to either single-agent modality, inhibiting tumor growth by 78% (*p* > 0.001, *n* = 7). Tumor regression or complete response was observed in 43% (3/7) of the mice in the combination arm, an outcome that was observed in just one other experimental animal—a member of the fenofibrate monotherapy group. Given the dramatic response of our xenograft tumors to fenofibrate, we speculated that fenofibrate might modulate a key tumor suppressor pathway, such as p53. To explore this possibility, we performed immunohistochemical analysis of p53 protein expression in our tumor sections. Compared to control, the number of p53+ stained tumor cells was significantly increased in all treatment groups. The control group showed, on average, 3.8 positive cells per high power field, compared to 24.3 in the fenofibrate treated condition (*p* < 0.01), 10 in the cisplatin group (*p* < 0.01), and 13.4 in the combination therapy group (*p* < 0.01) (Figure 6B). Consistent with this finding, a striking dose-dependent increase in p53 levels was observed in UMSCC47 and CaSki cells in response to fenofibrate treatment (Figure 6C).

To further explore tissue-level effects of fenofibrate and cisplatin treatments, hematoxylin- and eosin-stained (H&E) tumor sections were examined by light microscopy. Tumor sections from the untreated mice showed solid nests and islands of malignant squamous cells interspersed with scant stroma (Figure 6D). Necrosis was observed; however, it was less prominent in the control group compared to each of the treatment groups, which featured extensive central necrosis (Figure 6D–F). In mice that received fenofibrate, either as monotherapy or in combination, a heavy inflammatory cell infiltrate was also observed among the nests of malignant cells. In several cases, the inflamed portion comprised most of the non-necrotic tumor mass, with only small foci of malignant cells remaining (Figure 6F). In one mouse treated with combination therapy, a complete cure was observed. A small mass excised from the tumor site at the completion of the experiment showed only inflamed fibrous connective tissue, with no evidence of malignancy (Figure 6G).

Since necrosis was a prominent histologic feature in tumors from fenofibrate-treated mice, we speculated that fenofibrate may potentiate hypoxic cell death by blunting the adaptive hypoxia-inducible factor 1α (HIF1α) response. Nuclear HIF1α levels were dramatically elevated when UMSCC47 cells were cultured under hypoxic conditions (1% O_2_) compared to normoxic conditions (20% O_2_) (Figure 6H). Fenofibrate abrogated the hypoxia-induced increase in nuclear HIF1α levels. Similarly, nuclear programmed cell death ligand 1 (PD-L1), a downstream target of HIF1α, showed a striking increase in hypoxia, which was abolished by concurrent fenofibrate exposure.

## 4. Discussion

Nucleotide-based strategies for manipulating specific genes of interest have been invaluable for elucidating the function of genes and their roles in disease pathogenesis. A variety of such strategies, including siRNA, shRNA, TALEN, and CRISPR have been used to achieve repression of HPV16E6E7 in HPV+ cancers of both cervical and head and neck origins. Our work is distinct among studies employing nucleotide-based approaches to repress HPV16E6E7. To our knowledge, we are the first group to use an inducible system to silence HPV16E6E7 which avoids the inadvertent selection and expansion of resistant populations over the course of establishing a stable knockdown cell line. Given the essential nature of HPV16E6E7 to HPV+ cancer cell survival, any surviving cells with stable repression are likely to have intrinsic or de novo genomic alterations, transcriptomic alterations, or other biological idiosyncrasies that confer resistance to HPV16E6E7 silencing. Using a doxycycline-inducible system allowed us to study the phenotypic and transcriptomic consequences of HPV16E6E7 silencing in HPV+ cancer cells with temporal resolution in a representative, unselected cell population. Our results showed that HPV16E6E7 knockdown is highly effective in suppressing the proliferative capacity of HPV+ carcinoma cells in vitro and in vivo. Cell cycle and p53 pathways were modulated, confirming target specificity and demonstrating the robustness of our inducible HPV16E6E7 repression system.

Our motivation for this study was to develop and use an inducible HPV16E6E7 silencing system to identify key HPV16E6E7-regulated downstream signaling pathways that can be modulated with available drugs or drug-like molecules. We selected two druggable pathways, Wnt and PPAR, for initial study and evaluated several drugs known to target these pathways with good specificity. Although several candidate drugs showed significant anti-cancer activity in our preliminary studies, we later focused on fenofibrate because of its excellent safety profile [32] and potential to modulate both PPAR and Wnt signaling [33]. We showed that fenofibrate phenocopied HPV16E6E7 repression and inhibited cell proliferation and clonogenic survival of HPV+ cancer cells. Furthermore, fenofibrate monotherapy was as active as single-agent cisplatin in retarding in vivo tumor growth. The combination of fenofibrate and cisplatin was highly efficacious and resulted in regression or complete response in almost half the animals. Our results provide compelling evidence to support an exploratory clinical trial to assess the combination of fenofibrate and cisplatin in the HPV+ cancer setting.

Although we anticipated that fenofibrate would show anti-cancer activity, we were pleasantly surprised by the magnitude of the observed effect. In our in vitro studies, fenofibrate had anti-tumor effects that were similar in magnitude to those seen with HPVE6E7 knockdown. Moreover, the anticancer activity observed in our xenograft model was comparable to that seen with cisplatin, which, despite serious associated toxicities, is currently the best chemotherapeutic option for treating HPV+ HNSCC patients. In addition to demonstrating the clinical potential of fenofibrate in this setting, our findings raised questions about its mechanism of action. It would seem unlikely that modulation of one or a few pathways downstream of major genetic drivers would elicit such dramatic anticancer effects, and so we wondered if we might be modulating the major tumor suppressors targeted by HPVE6E7 more directly. Fenofibrate did not modulate pRb levels (data not shown) but did show an impressive recovery of p53 protein. The pharmacologic reactivation of p53 protein provides a likely explanation for the ability of fenofibrate to closely phenocopy HPVE6E7 knockdown. The biological mechanisms responsible for p53 accumulation in response to fenofibrate are unclear and certainly should be a focus of future research efforts.

Another intriguing finding is that systemic fenofibrate treatment resulted in a dramatic increase in immune cell infiltration. This infiltrate was associated with significant tumor destruction, suggestive of a functional, activated immune state within the tumor microenvironment. Our experiment utilized an athymic nude mouse model, which is expected to produce only immature T-cell precursors, natural killer cells, and macrophages [34], and thus the identity and functionality of the infiltrating immune cells is unclear. Since fenofibrate is able to promote a robust immune stimulatory effect in this immune-compromised model, we speculate that its immune-modulatory actions would be even greater in immune-competent model systems. Immune checkpoints, such as programmed death receptor 1/programmed death ligand 1 (PD-1/PD-L1), negatively modulate the density and phenotype of infiltrating immune cells and have become an important target for immunotherapies. PD-L1 expression on tumor cells is regulated in part by HIF1α, which binds directly to a hypoxia response element in its proximal promoter [35]. Given the large areas of central necrosis seen in tumor sections from treated mice, we speculated that fenofibrate might potentiate hypoxic cell death through modulation of HIF1α. Although fenofibrate did not affect HIF1α or PD-L1 levels in whole cell lysates from either normoxic or hypoxic conditions, nuclear fractions showed a dramatic increase in PD-L1 in hypoxia, which was abolished by concurrent treatment with fenofibrate. As reported by Gao and colleagues, nuclear PD-L1 modulates multiple immune response pathways to suppress immune cell activation [36]. In our study, fenofibrate suppressed the hypoxic increase in nuclear PD-L1 levels, and this action could contribute to the increased immune response seen in tumors from fenofibrate-treated mice. Additionally, PD-L1 is negatively regulated by wildtype p53 [37], so fenofibrate reprogramming of the tumor-immune microenvironment may be a direct consequence of reactivation of the p53 tumor suppressor program. Blockade of nuclear translocation of PD-L1 has been shown to enhance the efficacy of anti-PD-1 antibody therapy [36] and thus, our work provides support that fenofibrate in combination with PD-1/PD-L1-targeted immunotherapies may have clinical utility in the HPV+ HNSCC setting.

Although the anti-hyperlipidemic activity of fenofibrate is understood to occur through PPARα, the mechanisms responsible for the drug’s potent anti-cancer activity in HPV+ HNSCC are unclear. We did not observe any alteration in PPARα protein over a range of fenofibrate doses in either UMSCC47 or CaSki cells (Figure S2). Moreover, pathway analysis of the RNA-Seq dataset from UMSCC47 cells treated with fenofibrate over a range of time points confirmed enhancement of the p53 signaling pathway but did not show a significant effect on PPARα signaling (Table S2). When administered orally, fenofibrate readily undergoes ester hydrolysis to fenofibric acid, which is believed to be the active metabolite responsible for its PPARα-mediated anti-hyperlipidemic effects. By contrast, in the in vitro setting, fenofibrate remains largely in its original, unmetabolized form. When we compared the effects of fenofibrate and fenofibric acid, the acid form of the drug had no detectable anti-proliferative activity in UMSCC47 cells (Figure S3). If the anti-cancer effects observed in vivo are similarly attributable to the unmetabolized drug form, modification of fenofibrate to reduce ester hydrolysis could further improve potency.

The potential of fenofibrate as a cancer therapeutic has been described in a variety of cancer settings and various mechanisms of action have been proposed. A review by Lian and colleagues [32] lists 25 in vitro studies of fenofibrate’s anti-cancer activity in 12 different cancer types, including two relating to HPV− HNSCC. Of these papers, 12 reported PPARα-independent mechanisms and nine described PPARα-dependent or partially dependent mechanisms. Of the two papers examining the drug’s mechanism in HPV− HNSCC, both highlighted a role for AMPK signaling but disagreed on whether the mechanism was PPARα-dependent [38] or independent [39]. To the best of our knowledge, we are the first group to characterize the anti-cancer potential of fenofibrate to reactivate p53 in HPV+ HNSCC. Much remains to be elucidated regarding the drug’s mechanism in this setting, however, our findings suggest exciting potential to restore p53 function and stimulate a robust anti-tumor immunity.

## 5. Conclusions

In summary, treatment with fenofibrate alone or in combination with cisplatin or anti-PD-1 would provide a means of targeting the unique biology of HPV+ HNSCC, while reducing the toxicity and morbidity associated with current standard of care treatments. Moreover, given its excellent safety record, fenofibrate offers the exciting potential for long-term use as a preventative agent for individuals at high risk for developing primary or recurrent HPV+ HNSCC.

## Data Availability

The data presented in this study are available in this article (and supplementary material).

## Supplementary Materials

The following are available online at https://www.mdpi.com/article/10.3390/cancers14020282/s1. Figure S1: PPAR-selective agonists and Wnt inhibitors inhibit cell proliferation in HPV16+ HNSCC, Figure S2: PPAR levels are unchanged following fenofibrate treatment in HPV16+ SCC, Figure S3: Fenofibric acid does not inhibit proliferation of UMSCC47 cells, Table S1: Enriched and depleted KEGG pathways in response to HPV16E6E7 knockdown in UMSCC47 cells, and Table S2: Enriched and depleted KEGG pathways in response to fenofibrate treatment in UMSCC47 cells. File S1: Original West Blot Figures.

## Abbreviations

CIC	cancer initiating cells
CRISPR	clustered regularly interspaced short palindrome repeats
DMEM	Dulbecco’s modified Eagle medium
E6AP	E6-associated protein
HIF1α	hypoxia inducible factor 1α
HNSCC	head and neck squamous cell carcinoma
HPV	human papillomavirus
PD-1	programmed death protein-1
PD-L1	programmed cell death ligand-1
PPAR	peroxisome proliferator-activated receptor
STR	short tandem repeat
TALEN	transcription activator-like effector nucleases
Wnt	wingless/integrated
