# Supplementary material for: Repositioning Fenofibrate to Reactivate p53 and Reprogram the Tumor-Immune Microenvironment in HPV+ Head and Neck Squamous Cell Carcinoma
Source: Cancers (Basel). 2022 Jan 7;14(2):282. doi: 10.3390/cancers14020282 (PMC8773501; doi:10.3390/cancers14020282)
Supplement: Supplementary file 1 [file cancers-14-00282-s001.zip › FigureS1-S3 and TableS1&S2.pdf]

Figure S1. PPARγ-selective agonist, rosiglitazone, and Wnt inhibitors, FH535 and C5, inhibit cell proliferation in HPV16+ HNSCC.

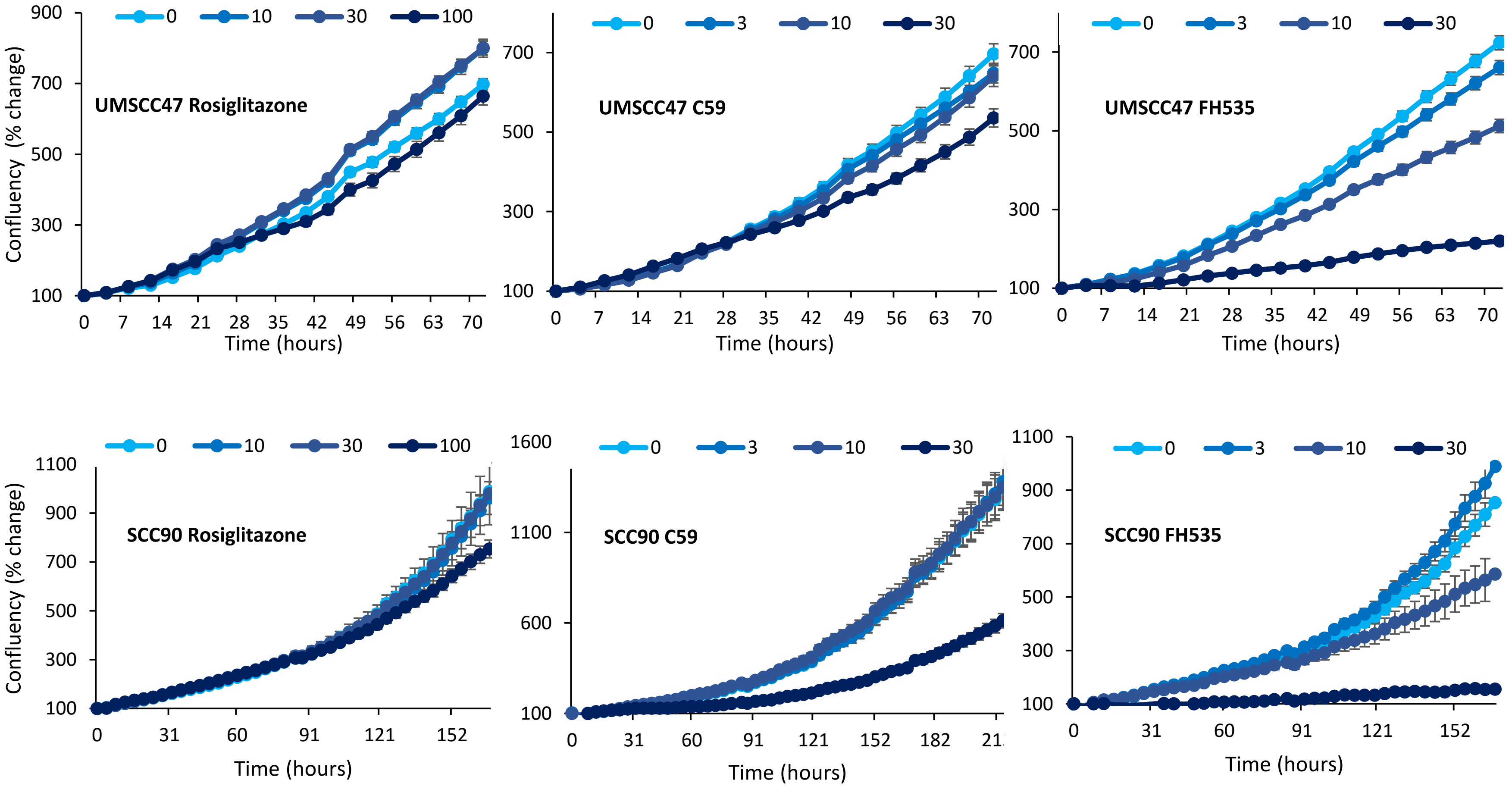

Figure S2. PPARα levels are unchanged following fenofibrate treatment in HPV16+ SCC.

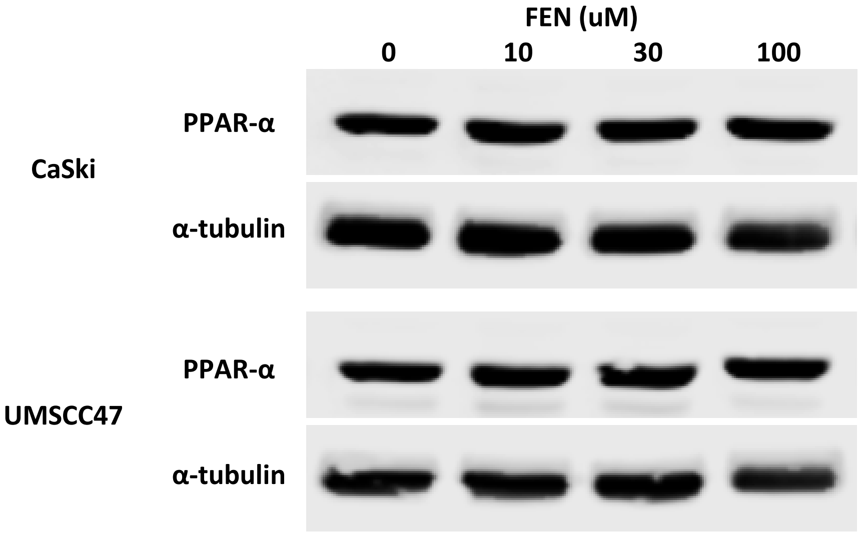

Figure S3. Fenofibric acid (FA) does not inhibit proliferation of UMSCC47 cells.

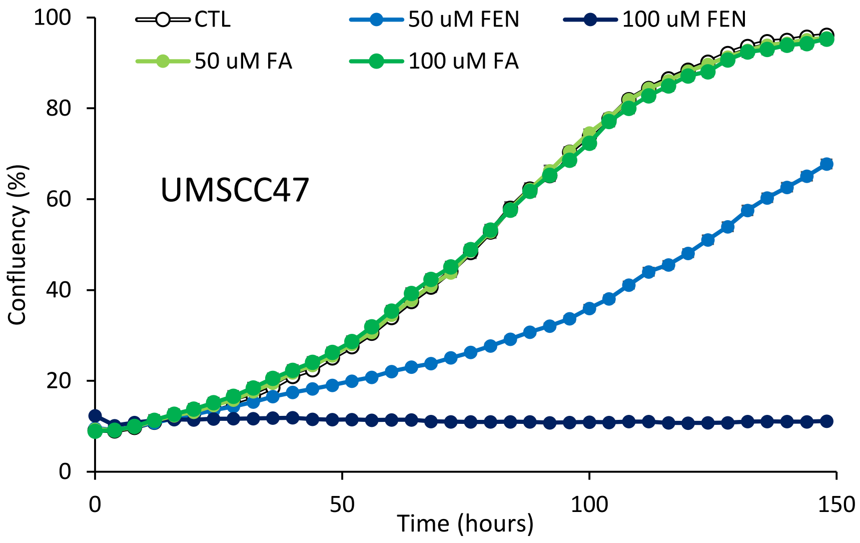

Table S1. Enriched and depleted KEGG pathways in response to HPV16E6E7 knockdown in UMSCC47 cells.

Enriched	FC	p-value	FDR	Depleted	FC	p-value	FDR
hsa03010 Ribosome	4.8	2.58E-16	4.23E-14	hsa04114 Oocyte meiosis	-3.8	3.23E-11	5.29E-09
hsa00190 Oxidative phosphorylation	3.8	6.81E-11	5.58E-09	hsa04110 Cell cycle	-3.7	1.37E-10	8.75E-09
hsa00980 Metabolism of xenobiotics by cytochrome P450	3.7	3.81E-10	2.08E-08	hsa04520 Adherens junction	-3.7	1.60E-10	8.75E-09
hsa00982 Drug metabolism - cytochrome P450	3.3	9.69E-09	3.97E-07	hsa04914 Progesterone-mediated oocyte maturation	-3.6	6.76E-10	2.77E-08
hsa04115 p53 signaling pathway	2.8	7.82E-07	2.57E-05	hsa03013 RNA transport	-3.2	2.03E-08	6.66E-07
hsa00280 Valine, leucine and isoleucine degradation	2.8	9.77E-07	2.67E-05	hsa04910 Insulin signaling pathway	-2.9	2.89E-07	7.90E-06
hsa00140 Steroid hormone biosynthesis	2.7	3.00E-06	6.56E-05	hsa04350 TGF-beta signaling pathway	-2.6	5.95E-06	0.000139
hsa00380 Tryptophan metabolism	2.7	3.20E-06	6.56E-05	hsa04144 Endocytosis	-2.4	2.33E-05	0.000477
hsa00640 Propanoate metabolism	2.7	4.06E-06	7.40E-05	hsa03015 mRNA surveillance pathway	-2.3	2.91E-05	0.000531
hsa00071 Fatty acid metabolism	2.5	1.47E-05	0.000241	hsa04540 Gap junction	-2.3	5.04E-05	0.000752
hsa04146 Peroxisome	2.3	2.95E-05	0.000439	hsa04810 Regulation of actin cytoskeleton	-2.2	5.45E-05	0.000752
hsa00650 Butanoate metabolism	2.4	3.89E-05	0.000532	hsa04320 Dorso-ventral axis formation	-2.3	5.51E-05	0.000752
hsa00590 Arachidonic acid metabolism	2.2	7.61E-05	0.000921	hsa03008 Ribosome biogenesis in eukaryotes	-2.2	0.000112	0.00141
hsa04610 Complement and coagulation cascades	2.2	7.86E-05	0.000921	hsa03040 Spliceosome	-2.1	0.000125	0.00146
hsa04974 Protein digestion and absorption	2.2	8.81E-05	0.000963	hsa04360 Axon guidance	-2	0.000283	0.003091
hsa04260 Cardiac muscle contraction	2.1	0.00013	0.001332	hsa00510 N-Glycan biosynthesis	-2	0.000316	0.003241
hsa00620 Pyruvate metabolism	2.1	0.000153	0.001477	hsa04310 Wnt signaling pathway	-2	0.000358	0.003457
hsa00340 Histidine metabolism	2.1	0.000252	0.0023	hsa04012 ErbB signaling pathway	-1.9	0.000514	0.004688
hsa00920 Sulfur metabolism	2.1	0.00028	0.002418	hsa04062 Chemokine signaling pathway	-1.8	0.001251	0.010796
hsa00480 Glutathione metabolism	1.9	0.000607	0.004974	hsa04010 MAPK signaling pathway	-1.7	0.001617	0.013261
hsa04623 Cytosolic DNA-sensing pathway	1.9	0.000637	0.004974	hsa03030 DNA replication	-1.7	0.00215	0.016789
hsa03320 PPAR signaling pathway	1.9	0.000721	0.005371	hsa04720 Long-term potentiation	-1.6	0.002792	0.020813

Table S2. Enriched and depleted KEGG pathways in response to fenofibrate treatment in UMSCC47 cells.

Pathway	DE genes	All genes	p-value
Ribosome	55	130	1.00E-24
Oxidative phosphorylation	31	106	1.35E-12
Alzheimer disease	32	140	1.4111E-06
Parkinson disease	32	111	1.9835E-06
Non-alcoholic fatty liver disease (NAFLD)	30	129	4.0637E-06
Huntington disease	36	156	5.2749E-06
Thermogenesis	38	183	7.0668E-06
Cardiac muscle contraction	11	39	3.6692E-05
Ribosome biogenesis in eukaryotes	15	71	6.4002E-05
Steroid biosynthesis	7	17	6.7204E-05
Retrograde endocannabinoid signaling	15	86	0.00160234
Mineral absorption	8	32	0.00248638
Proteasome	8	42	0.00637399
Cytokine-cytokine receptor interaction	13	109	0.00743111
Spliceosome	16	128	0.01244393
p53 signaling pathway	11	64	0.01639304
Salmonella infection	10	71	0.01716173
Metabolic pathways	86	1029	0.02190162
Parathyroid hormone synthesis, secretion and action	11	78	0.04683979
