# Supplementary material for: Repositioning Fenofibrate to Reactivate p53 and Reprogram the Tumor-Immune Microenvironment in HPV+ Head and Neck Squamous Cell Carcinoma
Source: Cancers (Basel). 2022 Jan 7;14(2):282. doi: 10.3390/cancers14020282 (PMC8773501; doi:10.3390/cancers14020282)
Supplement: Supplementary file 1 [file cancers-14-00282-s001.zip › File S1.pdf]

Supplemental Data: Blots with Densitometry

Figure 1B

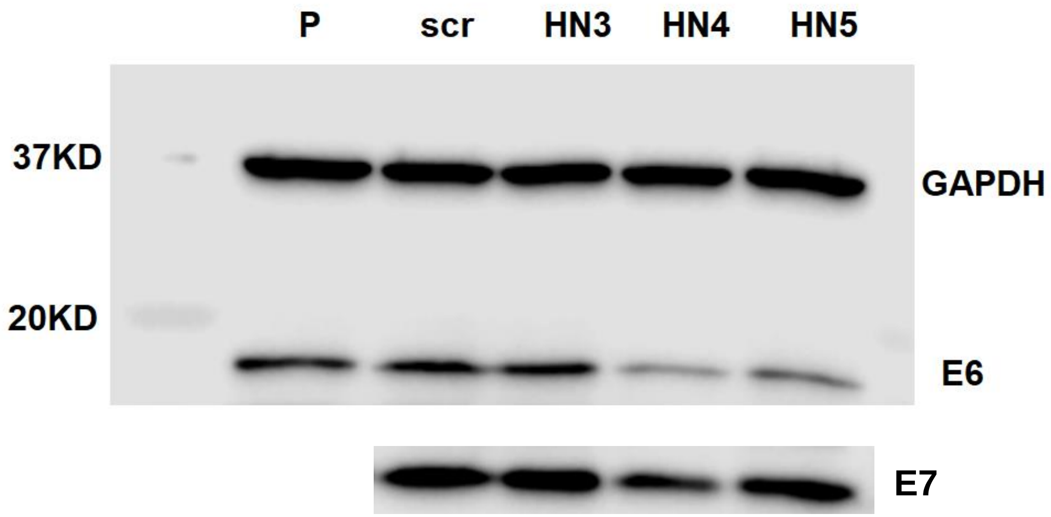

Figure 1B										
	CTL (scr)	shRNA3	shRNA4	shRNA5			CTL (scr)	shRNA3	shRNA4	shRNA5
E6	31466	30960	19066	22606		E6/GAPDH	0.592468	0.615336	0.34612	0.37761
E7	54480	54289	40186	58972		E7/GAPDH	1.025796	1.079004	0.729527	0.985067
GAPDH	53110	50314	55085	59866						
						E6/GAPDH	1	1.038597	0.584199	0.63735
						E7/GAPDH	1	1.05187	0.711182	0.960295

Figure 6C

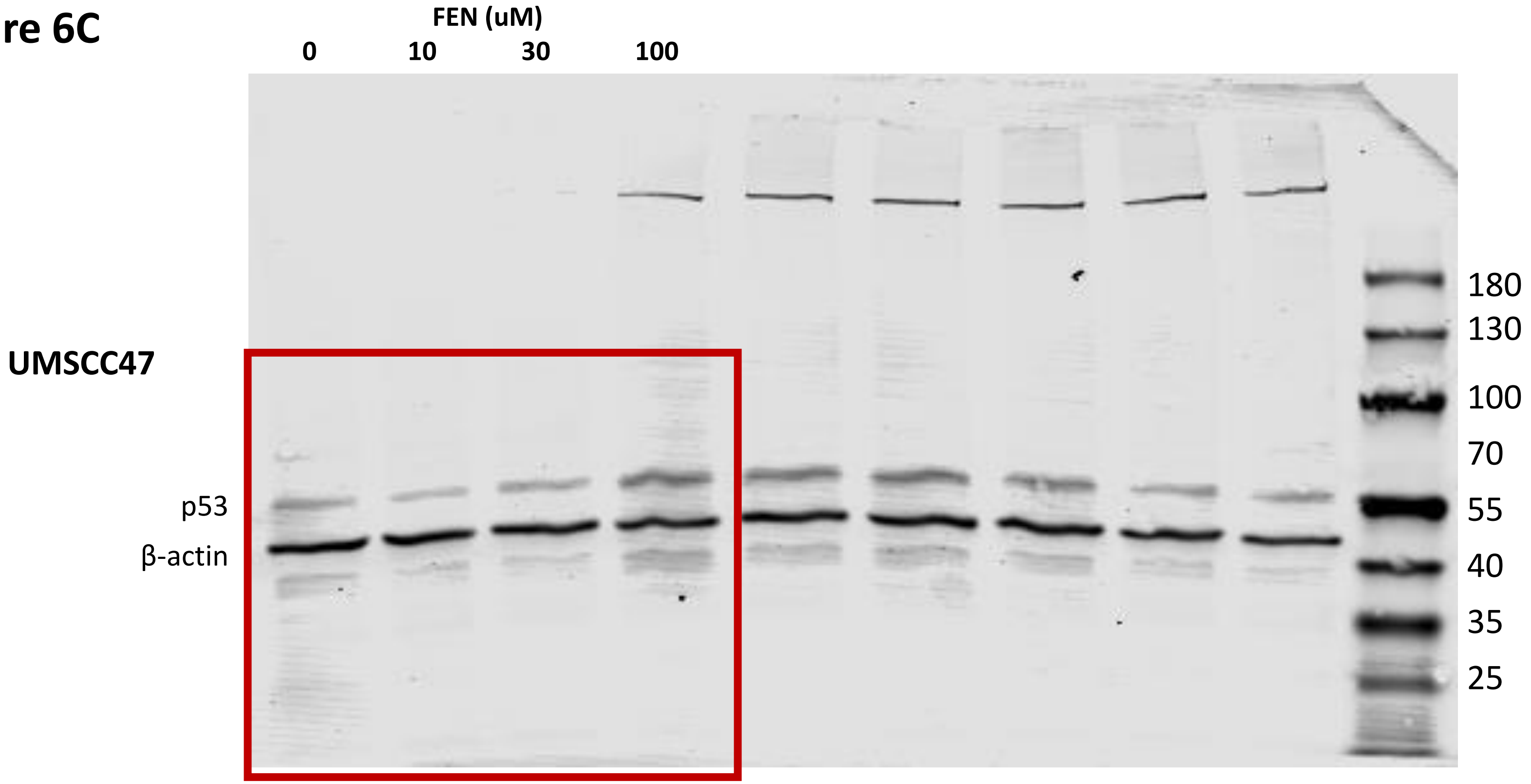

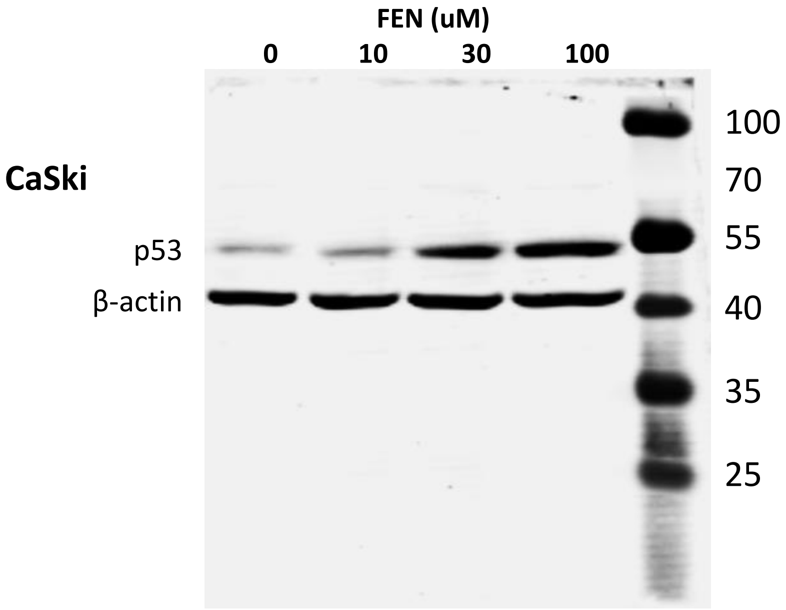

	FEN (uM)			
**UMSCC47**	0	10	30	100
p53	308	261	383	504
β-actin	19900	17700	19400	17800
p53/β-actin	0.01548	0.01475	0.01974	0.02832
p53/β-actin	1	1.23502	2.65714	4.52987
**CaSki**				
p53	238	268	651	1090
β-actin	27200	24800	28000	27500
p53/β-actin	0.00875	0.01081	0.02325	0.03964
p53/β-actin	1	1.23502	2.65714	4.52987

Figure 6E

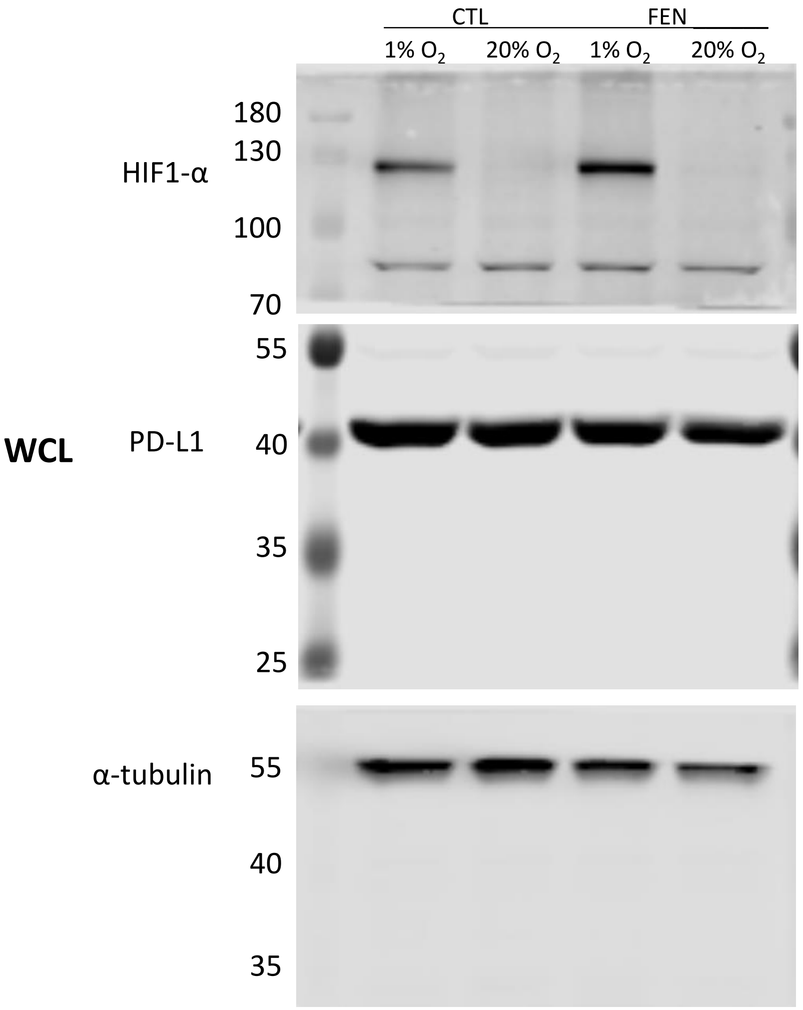

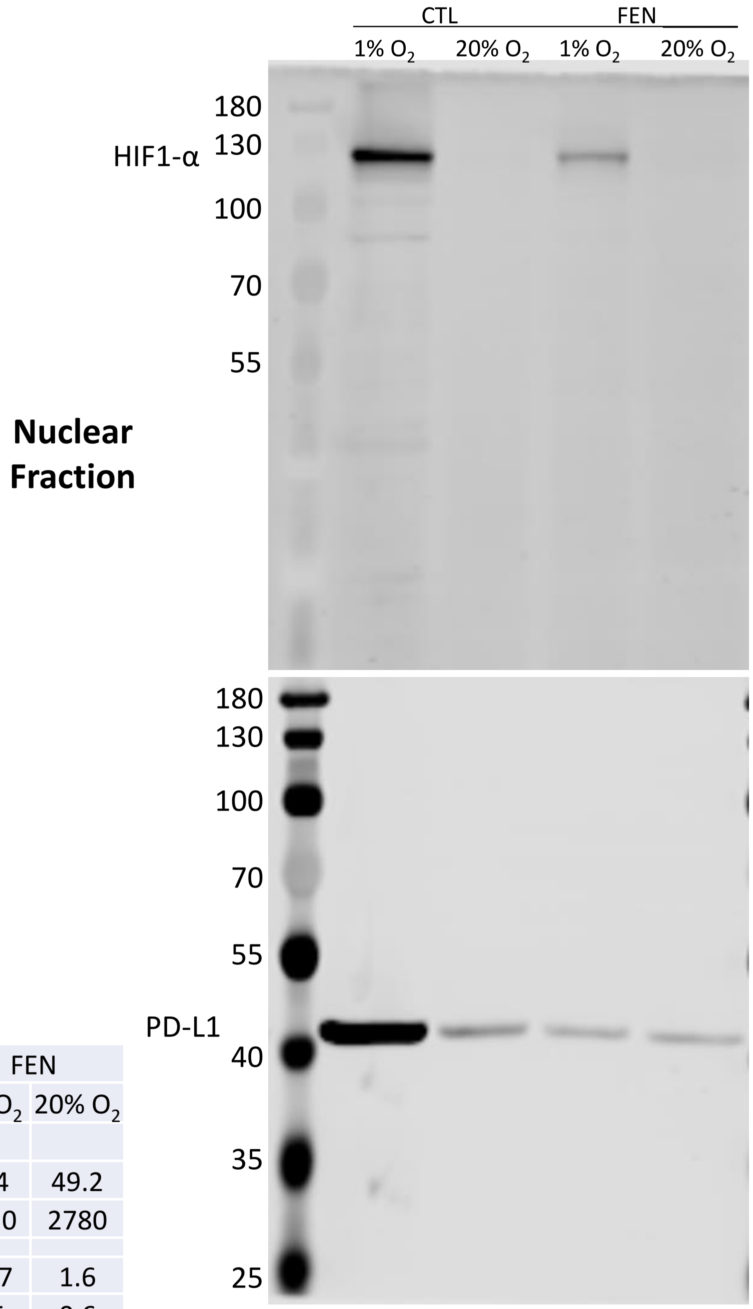

	CTL		FEN				CTL		FEN	
**WCL**	1% O_2	20% O_2	1% O_2	20% O_2		**Nuclear Fraction**	1% O_2	20% O_2	1% O_2	20% O_2
HIF1-α	1220	80.6	1960	62.6						
PD-L1	101000	84400	82600	73100		HIF1-α	3580	30.5	784	49.2
α-tubulin	44400	43600	31700	25000		PD-L1	75000	4860	2530	2780
HIF1-α/α-tubulin	0.027	0.002	0.062	0.003		HIF1-α	117.4	1	25.7	1.6
PD-L1/α-tubulin	2.275	1.936	2.606	2.924		PD-L1	15.4	1	0.5	0.6
HIF1-α/α-tubulin	14.9	1	33.4	1.4						
PD-L1/α-tubulin	1.2	1	1.3	1.5						

Figure S2

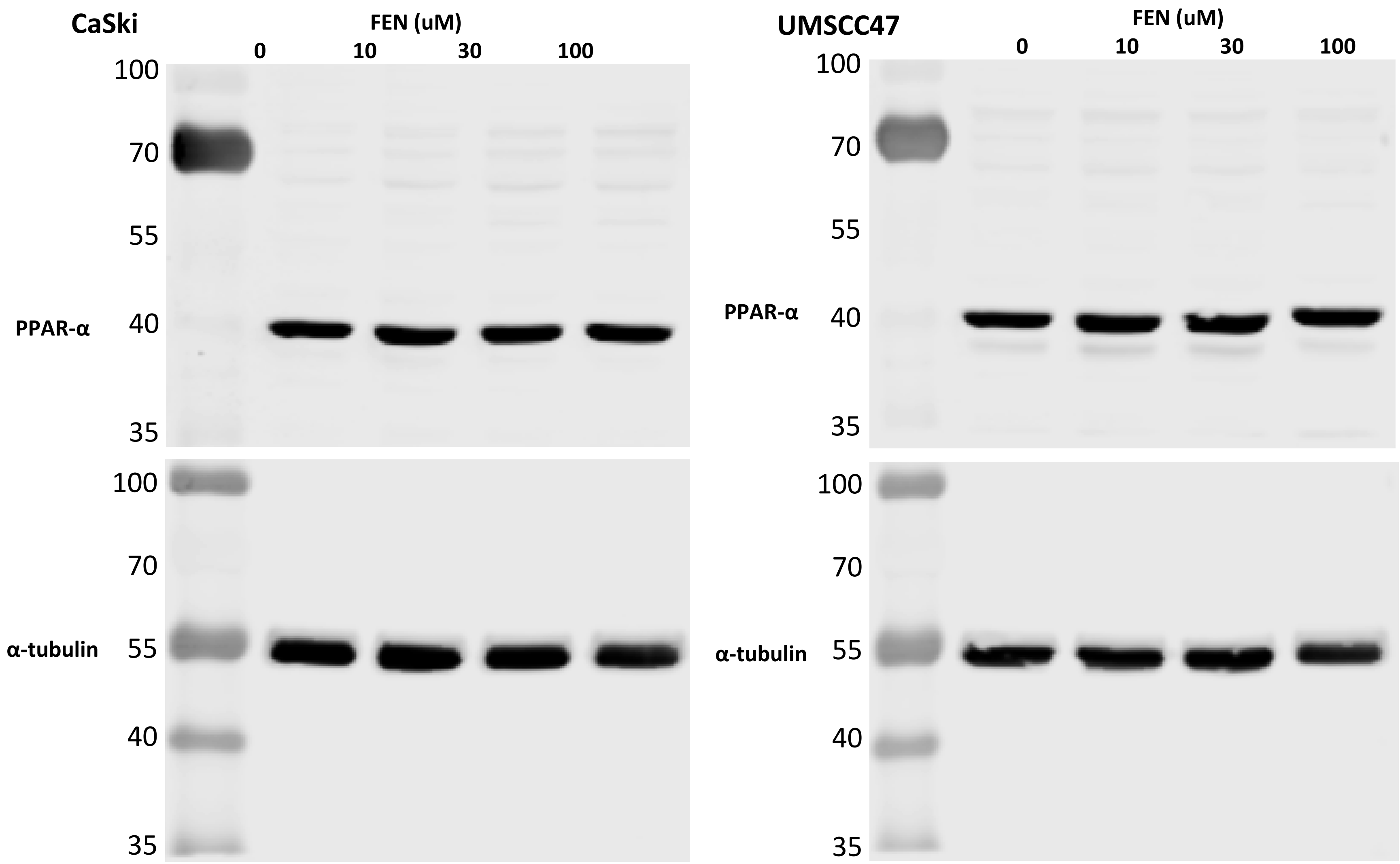

FIG S2	FEN (uM)						FEN (uM)			
**CaSki**	0	10	30	100		**UMSCC47**	0	10	30	100
PPAR-α	256000	275000	269000	284000		PPAR-α	270000	301000	293000	297000
α-tubulin	398000	409000	385000	348000		α-tubulin	317000	337000	354000	299000
PPAR-α/α-tubulin	0.64322	0.67237	0.69870	0.81609		PPAR-α/α-tubulin	0.85174	0.89318	0.82768	0.99331
PPAR-α/α-tubulin	1	1.04533	1.08626	1.26877		PPAR-α/α-tubulin	1	1.04865	0.97176	1.16622
